# The E3 ubiquitin ligase SCF(Fbxo7) mediates proteasomal degradation of UXT isoform 2 (UXT-V2) to inhibit the NF-κB signaling pathway

**DOI:** 10.1016/j.bbagen.2020.129754

**Published:** 2021-01

**Authors:** Valentine Spagnol, Caio A.B. Oliveira, Suzanne J. Randle, Patrícia M.S. Passos, Camila R.S.T.B. Correia, Natália B. Simaroli, Joice S. Oliveira, Tycho E.T. Mevissen, Ana Carla Medeiros, Marcelo D. Gomes, David Komander, Heike Laman, Felipe Roberti Teixeira

**Affiliations:** a Department of Genetics and Evolution, Federal University of Sao Carlos, Brazil; b Department of Biochemistry and Immunology, Ribeirao Preto Medical School, University of Sao Paulo, Brazil; c Department of Pathology, University of Cambridge, Tennis Court Road, Cambridge CB2 1QP, UK; d MRC Laboratory of Molecular Biology, Francis Crick Avenue, Cambridge Biomedical Campus, Cambridge CB2 0QH, UK; e Harvard Medical School, Department of Biological Chemistry and Molecular Pharmacology, 250 Longwood Ave, Boston, MA 02115, USA; f The Walter and Eliza Hall Institute of Medical Research, Ubiquitin Signalling Division, 1G Royal Parade, Parkville 3052, VIC, Australia

**Keywords:** E3 ubiquitin ligase, NF-kappa B (NF-κB), SCF(Fbxo7), UXT-V2, UXT-V1, Ubiquitylation (ubiquitination)

## Abstract

**Background:**

Ubiquitously eXpressed Transcript isoform 2 (UXT—V2) is a prefoldin-like protein involved in NF-κB signaling, apoptosis, and the androgen and estrogen response. UXT-V2 is a cofactor in the NF-κB transcriptional enhanceosome, and its knockdown inhibits TNF-α -induced NF-κB activation. Fbxo7 is an F-box protein that interacts with SKP1, Cullin1 and RBX1 proteins to form an SCF(Fbxo7) E3 ubiquitin ligase complex. Fbxo7 negatively regulates NF-κB signaling through TRAF2 and cIAP1 ubiquitination.

**Methods:**

We combine co-immunoprecipitation, ubiquitination *in vitro* and *in vivo*, cycloheximide chase assay, ubiquitin chain restriction analysis and microscopy to investigate interaction between Fbxo7 and overexpressed UXT-V2-HA.

**Results:**

The Ubl domain of Fbxo7 contributes to interaction with UXT—V2. This substrate is polyubiquitinated by SCF(Fbxo7) with K48 and K63 ubiquitin chain linkages *in vitro* and *in vivo*. This post-translational modification decreases UXT-V2 stability and promotes its proteasomal degradation. We further show that UXT—V1, an alternatively spliced isoform of UXT, containing 12 additional amino acids at the N-terminus as compared to UXT—V2, also interacts with and is ubiquitinated by Fbxo7. Moreover, *FBXO7* knockdown promotes UXT-V2 accumulation, and the overexpression of Fbxo7-ΔF-box protects UXT-V2 from proteasomal degradation and enhances the responsiveness of NF-κB reporter. We find that UXT-V2 colocalizes with Fbxo7 in the cell nucleus.

**Conclusions:**

Together, our study reveals that SCF(Fbxo7) mediates the proteasomal degradation of UXT-V2 causing the inhibition of the NF-κB signaling pathway.

**General significance:**

Discovering new substrates of E3 ubiquitin-ligase SCF(Fbxo7) contributes to understand its function in different diseases such as cancer and Parkinson.

## Highlights

• UXT-V2 is a canonical substrate of SCF(Fbxo7) E3 ubiquitin ligase.
• Fbxo7 interacts with both UXT-V1 and UXT—V2.
• UXT-V2 recruits Fbxo7 to the cell nuclei.
• Fbxo7 inhibit NF-kB pathway through degradation of UXT-V2.

## 1 Introduction

F-box proteins (FBPs) are components of the largest family of SCF-type (SKP1, Cullin1 and F-box protein) E3 ubiquitin ligases called SCF1 (SKP1, Cullin1 and F-box protein) or CRLs (Cullin-RING ligases) [1]. FBPs interact through their F-box domain with SKP1, which binds to a scaffold protein Cullin 1 and RING-box protein RBX1 to form E3 ligase complexes, which are the final players in the enzymatic cascade responsible for protein modification with ubiquitin [[2], [3], [4]]. This reversible process is catalyzed by three different enzymes: a ubiquitin-activating enzyme (E1), a ubiquitin-conjugating enzyme (E2), and a ubiquitin ligase (E3). The consequences of this post-translational modification (PTM) include proteasomal degradation [5] and/or altering the localization or activity of the modified protein [6]. Opposing the effect of E3 ubiquitin ligases, deubiquitinating enzymes (DUBs) catalyze the removal of ubiquitin from target proteins and disassemble polymeric ubiquitin chains, thus changing the fate of substrates and maintaining ubiquitin homeostasis in the cell [7].

The dysregulation of FBPs causes a range of different human pathologies, such as cancer (reviewed in [8,9]), Parkinson's disease (PD) [10] and cardiac diseases [11], attesting to their importance in the regulation of many cellular processes such as the cell cycle, differentiation and development, cell death, and oxidative stress [12]. Fbxo7 is the fifth most abundant F-box protein found in SCF complexes in human cells [13]; however, many of its described functions, such as cell cycle regulation [14,15] and mitophagy, are independent of its SCF(Fbxo7) ubiquitin ligase activity [16]. The effects of Fbxo7 on the cell cycle are mediated through its stabilizing interactions with cell cycle proteins, Cdk6 and p27 [14,17]. Recessive point mutations in *FBXO7*, also called *PARK15*, are associated with PD [[18], [19], [20], [21]], and deficiencies in mitochondrial homeostasis are emerging as a possible cause [16,21,22]. Fbxo7, but not its PD mutants, facilitates mitophagy to protect cells from accumulating damaged mitochondria, and upon neurotoxic stress, Fbxo7 localizes in mitochondria and forms aggregates. Fbxo7 mutations promote deleterious Fbxo7 aggregation in mitochondria, contributing to cell death [22]. SCF(Fbxo7) inhibits the NF-κB (nuclear factor-κB) signaling pathway through ubiquitination of c-IAP1 (inhibitor of apoptosis) and TRAF2 [[23], [24]]. The neurotrophin receptor-interacting MAGE protein (NRAGE), a component of bone morphogenetic protein receptor (BMPR) signaling with XIAP–TAK1–TAB1 complex, is also poly-ubiquitinated by Fbxo7 up-regulating the NF-κB activity [25]. Other ubiquitinated substrates include translocase of outer mitochondrial membrane 20 (TOMM20) and glycogen synthase kinase 3β (GSK3β) which were validated as K63-polyubiquitinated substrates, suggesting Fbxo7 plays roles in mitochondrial transport and Wnt signaling pathway regulation [26].

From the same proteome-wide screen that discovered TOMM20 and GSK3β as substrates of SCF(Fbxo7), we also identified Ubiquitously eXpressed Transcript (UXT) protein isoform 2, also known as androgen receptor trapped clone-27 (ART-27). UXT is a prefoldin-like protein that forms ubiquitously expressed protein-folding complexes in human and mouse tissues. Two mRNA splicing isoforms of UXT, termed UXT-V1 and UXT—V2, have been described. They differ in their N-termini, (UXT-V1 (169 aa) is 12 amino acids longer than UXT-V2 (157 aa) and have distinct functions. While UXT-V2 is a transcriptional cofactor that activates NF-κB transcription in the nucleus, UXT-V1 is localized in the cytoplasm and modulates TNF-induced apoptosis [27,28]. UXT-V2 also regulates the androgen receptor signaling pathway through interactions with Androgen Receptor N-terminus [29,30]. In addition, these isoforms have opposing effects in SARM (Sterile and HEAT ARMadillo motif-containing protein)-induced apoptosis, with UXT-V1 promoting a reduction in caspase 8 activity and UXT-V2 increasing caspase 8 activity and enhancing apoptosis by activating the extrinsic pathway through depolarization of mitochondria [31]. UXT-V2 is also implicated in tumorigenesis, being overexpressed in a number of human tumors but not in matching normal tissues. In the cytoplasm, EGFP-UXT-V2 has been found associated with γ-tubulin in human centrosomes, and it causes mitochondrial aggregation when overexpressed [32,33].

Although Fbxo7 and UXT-V2 are involved in similar cellular processes, such as NF-κB signaling and stress-induced mitochondrial aggregation, the interaction between these proteins has not been explored. Here, we show that Fbxo7 interacts and ubiquitinates both UXT-V1 and UXT—V2, with UXT-V2 being poly-ubiquitinated through K48 and K63 polyubiquitin chains. This modification reduces UXT-V2 stability, inducing proteasomal degradation. We show that UXT-V2 colocalizes with Fbxo7 in the cell nucleus. The knockdown of *FBXO7* or the overexpression of the dominant negative form of Fbxo7, called Fbxo7-ΔF-box, increases UXT-V2-HA protein levels. Finally, the overexpression of Fbxo7-ΔF-box, increases TNF-α activation of the NF-κB signaling pathway.

## 2 Material and methods

### 2.1 Cell culture

Human osteosarcoma epithelial cells (U2OS) and human embryonic kidney (HEK) 293 T cells were obtained from ATCC. The cells were cultured in DMEM high glucose (Corning) supplemented with 10% fetal bovine serum (FBS, Gibco) and penicillin (100 units), streptomycin (100 μg) and l-glutamine (0.292 mg/mL) (Thermo Fisher Scientific). To passage cells, they were washed once with phosphate buffered saline 1× (PBS, HyClone) and detached with trypsin (TrypLe Express, Thermo Fisher Scientific).

### 2.2 Reagents and antibodies

Cycloheximide (C1988), protease inhibitor cocktail SIGMAFAST™ (S8820), agarose- anti-FLAG® M2 (A2220), agarose-anti-HA (E6779) beads, FLAG® peptide (F3290), HA peptide (I2149), primers were all purchased from Sigma-Aldrich. Antibodies to HA (H3663) (1:1000), FLAG® M2 (F1804) (1:500), Fbxo7 (SAB1407251) (1:1000), GAPDH (G8795) (1:10000) and actin (A3853) (1:2000) were purchased from Sigma-Aldrich. Rabbit antibodies to Fbxo7 (ARP43128) (1:1000) were purchased from Aviva Systems Biology; antibodies against β-actin were purchased from Merck Millipore (MAB1501) (1:10000); and against the myc epitope (#2272) (1:1000), anti-K63 polyubiquitin (#5621) (1:500), anti-K48 polyubiquitin (#8081) (1:1000), anti-AKT (#4691) (1:1000) anti- Histone H3 (1B1B2) (1:1000) were purchased from Cell Signaling Technologies. Human ubiquitin (U—100H), ubiquitin N-terminal biotin (UB-560), His-ubiquitin E1 enzyme (UBE1) (E-304), UbcH5a/UBE2D1 (E2-616), 10× ubiquitin conjugation reaction buffer (B-70), Mg-ATP Solution (B-20) and the proteasome inhibitor MG132 (I-130) were purchased from Boston Biochem.

### 2.3 Plasmids and cloning

Constructs expressing wild-type or mutant Fbxo7 cloned into pcDNA3 or empty vector (EV), as well as Ub-myc-6xHis, have been previously described [26]. pEGFP-N1 was obtained from Clontech. The pcDNA3-UXT-V2-HA plasmid was constructed from pGEX2-UXT (kindly provided by Dr. Chris Bartholomew, Glasgow Caledonian University) through PCR using the following primers: sense (*Eco*RI site) 5’ CTACGGGAATTCATGGCGACGCCCCCTAAGCGGC 3′ and antisense (*Xho*I site) 5’ACCGAGCTCGAGCTAAGCGTAATCTGGTACGTCGTATGGGTAATGGTGAGGCTTCTCTGGGAAATTCTGCAG 3′. This construct was used as a template for the construction of UXT-V1-HA by PCR. The extra 12 N-terminal amino acid sequence of UXT-V1 was added to the sense primer, while the HA-tag encoding sequence of UXT-V2-HA was added to the antisense primer. For PCR amplification, 67.5 ng of the sense primer (*Eco*RI site) 5’ATACTAGAATTCATGGTCTTCCCCCTCCCCACTCCCCAGGAGCCCATCATGGCGACGCCCCCTAAGC 3′ and antisense primer (*Xho*I site) 5’ TATGAGCTCGAGCTAAGCGTAATCTGGTACGTCGTATGGGTAATGG 3′ was used with 10 ng of the template and Phusion Flash High-Fidelity PCR Master Mix (Thermo Scientific, USA). The pcDNA3-UXT-V1-M13G-HA plasmid was purchased from Epoch Life Science (USA). The siRNA to human *FBXO7* 5’CUGAGUCAAUUCAAGAUAA3’ was obtained from Sigma-Aldrich.

### 2.4 *In vitro* ubiquitination assays

For the *in vitro* ubiquitination assay, the SCF(Fbxo7) complex and Fbxo7 lacking the F-box domain (Fbxo7-ΔF-box) were purified from HEK293T cells transfected with 2xFLAG-Fbxo7 or 2xFLAG-Fbxo7-ΔF-box in combination with SKP1-HA, RBX1-myc and Cullin1. The cells were lysed with NP-40 lysis buffer (50 mM Tris-HCl pH 7.2, 225 mM KCl, and 1% NP-40) supplemented with a protease inhibitor cocktail and phosphatase inhibitors (10 mM NaF and 1 mM Na_3_VO_4_). Cell lysates were centrifuged 16,900 x*g*/4 °C/20 min and supernatants were subjected to immunoprecipitation (IP) with anti-FLAG agarose beads, and the eluates were obtained after elution with FLAG peptide (300 μg/mL) during 1 h at 4 °C. SCF(Fbxo7) or Fbxo7-ΔF-box was used at 25 and 50 nM combined with the ubiquitin mix [ubiquitin buffer, E1 (100 nM), E2-UbcH5a (500 nM), biotin-ubiquitin (20 mM), Mg-ATP (2 mM)] and UXT-V2-HA purified from HEK293T. The reactions were incubated at 30 °C for 90 min and resolved by SDS-PAGE for immunoblotting assays.

### 2.5 *In vivo* ubiquitination assays

HEK293T cells were transfected with empty vector, EGFP or FLAG-Fbxo7 constructs and UXT-V2-HA or UXT-V1-M13G-HA, with or without ubiquitin-6xHis-myc for 36 h. The transfected cells were treated with 10 μM of MG132 6 h prior to lysis. Cells were lysed as described in last section and supernatants were subjected to immunoprecipitation (IP) with agarose-anti-HA beads. The polyubiquitinated proteins were eluted with HA peptide (300 μg/mL), and eluates were resolved with SDS-PAGE and immunoblotted. To reprobe immunoblots, membranes were incubated in a Stripping Buffer (SB) (glycine 200 mM pH 2.0, SDS 0.1%, Tween 1%) at 37 °C for 15 min. This incubation was repeated 3 times, and the membrane was washed with TBST and blocked for 1 h with dried milk 5% in TBST. Thereafter, the membrane was tested with anti-rabbit-HRP antibody to ensure the removal of previous signals. The second probe with rabbit anti-K48 antibody was then performed. The images were captured in ChemiDoc XRS+ (BioRad), one image each 4 s during 200 s, before pixel saturation and directly used for analysis.

### 2.6 Ubiquitin chain restriction analysis

Ubiquitin chain restriction (UbiCRest) analyses were performed as described [26,34]. HEK293T cells were transfected for 48 h with UXT-V2-HA and 2xFLAG-Fbxo7 and treated 6 h prior to cell lysis with MG132 (10 μM). The polyubiquitinated UXT-V2-HA were immunoprecipitated from HEK293T cells using agarose-anti-HA. The *in vivo* polyubiquitinated substrates were eluted by HA peptide at 300 μg/mL and stored at −80 °C. Purified deubiquitinating enzymes (DUBs) as described in [34] were diluted with 2× dilution buffer (50 mM Tris, pH 7.4, 300 mM NaCl, and 20 mM DTT), added to the samples for 30 min at 37 °C, and reactions were stopped by Laemmli buffer. Samples were resolved by SDS–PAGE, and the blots were probed with anti-polyubiquitin (Santa Cruz Biotechnology, CA, USA).

### 2.7 Cycloheximide chase assay

U2OS cells were transfected with 2xFLAG-Fbxo7 or 2xFLAG-Fbxo7-ΔF-box in combination with UXT-V2-HA or UXT-V1-M13G-HA. Cells were serum starved for 12 h and, where indicated, treated with cycloheximide (40 μg/mL) for 0, 2 or 4 h in the presence or absence of the proteasome inhibitor MG132 (10 μM, 4 h) prior to lysis. Cell lysis was performed with RIPA buffer (25 mM Tris-HCl pH 7.6, 150 mM NaCl, 1% NP-40, 1% sodium deoxycholate and 1% sodium dodecyl sulfate) with a protease inhibitor cocktail and phosphatase inhibitors as previously described for 30 min on ice. Solutions were centrifuged at 16,900 x*g* for 45 min at 4 °C, and supernatants containing protein extracts were submitted to SDS-PAGE and immunoblotting.

### 2.8 *FBXO7* knockdown

HEK293T cells were transfected with 100 nM of *FBXO7*- siRNA or siRNA universal negative control (Sigma Aldrich) in the presence of 0,5 μg of UXT-V2-HA by using Lipofectamine 2000, according to the manufacturer's instructions (Thermo Scientific, USA). After 36 h, cells were lysates with RIPA buffer as described before and the protein extracts were used to immunoblotting.

### 2.9 Reverse transcriptase-quantitative PCR (RT-qPCR) analysis

Total RNA was extracted from HEK293T cells transfected with pEGFP-N1, pcDNA3 or UXT-V2 with the Direct-zol RNA MiniPrep kit (Zymo Research, USA) and treated with DNAse I (Invitrogen™, USA) for DNA removal according to the manufacturer's instructions. Samples were quantified with NanoVue™ (GE Healthcare Life Sciences, USA), and 300 ng of RNA was reverse transcribed with a High Capacity cDNA Reverse Transcription Kit (Thermo Fisher Scientific, USA). cDNA samples were diluted 25× for use as a template, and primers were used at 150 nM in a 15 μL reactions with SYBR™ Green PCR Master Mix (Applied Biosystems™, USA). Amplifications were carried out in a 7500 Fast Real-Time PCR System (Applied Biosystems™, USA) with the following primers: *FBXO7* sense: 5’ AGTCCCTGCTGTGCACCTG 3′, *FBXO7* antisense: 5’ CGCTGGAATGTCATCTTGAAGA 3′, *GAPDH* sense: 5’ AGAAGGCTGGGGCTCATTTG 3′, and *GAPDH* antisense: 5’ AGGGGCCATCCACAGTCTTC 3′.

### 2.10 Preparation of total and subcellular extracts

Total levels of Fbxo7 were assessed by transfecting HEK293T cells with pEGFP-N1 or UXT—V2, which were lysed with 2× Laemmli sample buffer and submitted to SDS-PAGE and immunoblotting. For subcellular fraction enrichment, U2OS cells were transfected with empty vector or UXT-V2-HA or UXT-V1-M13G-HA in combination with 2xFLAG-Fbxo7 and lysed with NE-PER® Nuclear and Cytoplasmic Extraction Reagents according to the manufacturer's protocol (Thermo Scientific, USA). Supernatants containing the protein extracts were submitted to SDS-PAGE and immunoblotting.

### 2.11 NF-κB luciferase reporter assay

HEK293 cells expressing pBIIx-luc (described in [35]) were kindly provided by Dr. Dario Zamboni (Department of Cellular and Molecular Biology, Ribeirao Preto Medical School, University of Sao Paulo). Cells were cultivated in 96-well Corning Costar® plates at 5 × 10^5^ cells/well, and after 24 h, they were transfected with 75 ng of each plasmid (pEGFP-N1, 2xFLAG-Fbxo7 or 2xFLAG-Fbxo7-ΔF-box or UXT-V2-HA) and 1,5 ng of pCMV-Renilla luciferase control plasmid, with Lipofectamine 2000 (Thermo Scientific). After 24 h, the cells were treated with TNF-α 10 ng/mL in serum deprived DMEM without phenol red (Gibco) by 12 h. Cells were lysed by Dual-Glo Luciferase assay kit (Promega), transferred to white plate 96-well Corning Costar® plate and Firefly and Renilla luciferase activity measurement were obtained in a SpectraMax i3 luminometer (Molecular Devices).

### 2.12 Multiphoton microscopy

U2OS cells were grown on glass coverslips in complete DMEM and transfected with UXT-V2-HA, UXT-V1-M13G-HA or 2xFLAG-Fbxo7 with Lipofectamine 2000 according to the manufacturer (Thermo Scientific, USA). The cells were washed once with PBS before being fixed and permeabilized for 10 min at room temperature (RT) with PBS containing 2% paraformaldehyde, 0.3% Triton X-100. Subsequently, cells were washed three times with PBS and blocked with PBS containing 2% bovine serum albumin (BSA) for 1 h at RT. Antibody incubations were performed for 1 h at RT in PBS containing 2% BSA (dilutions: anti-Fbxo7 (Aviva) 1:50; anti-HA (Sigma Aldrich) 1:600) followed by incubation with Alexa 488- and Alexa 594-coupled secondary antibodies (Thermo Scientific, USA) (dilution 1:800). Coverslips were mounted with Prolong diamond antifade mounting medium containing DAPI (Invitrogen). Samples were analyzed with the multiphoton laser scanning microscope Zeiss LSM 780 (Carl Zeiss) using objective C-Apochromatic 63×/1.2 W in Laboratório Multiusuário de Microscopia Multifoton (LMMM) at the Department of Cellular and Molecular Biology, Ribeirao Preto Medical School, University of Sao Paulo, Brazil.

### 2.13 Statistical analysis

Data were subjected either to unpaired Student's *t*-test (when there were only two groups) or one-way analysis of variance (ANOVA one-way) or 2way ANOVA with Bonferroni or Newman-Keuls pottest. Analyses were performed with Prism (GraphPad Software, USA), and statistical significance was accepted at *p-values* ≤ 0.05*: *p* ≤ 0.01** and: *p* ≤ 0.001***.

## 3 Results

### 3.1 SCF(Fbxo7) ubiquitinates UXT-V2 in vitro and interacts with UXT-V1 and UXT—V2

**Fig. 1:**
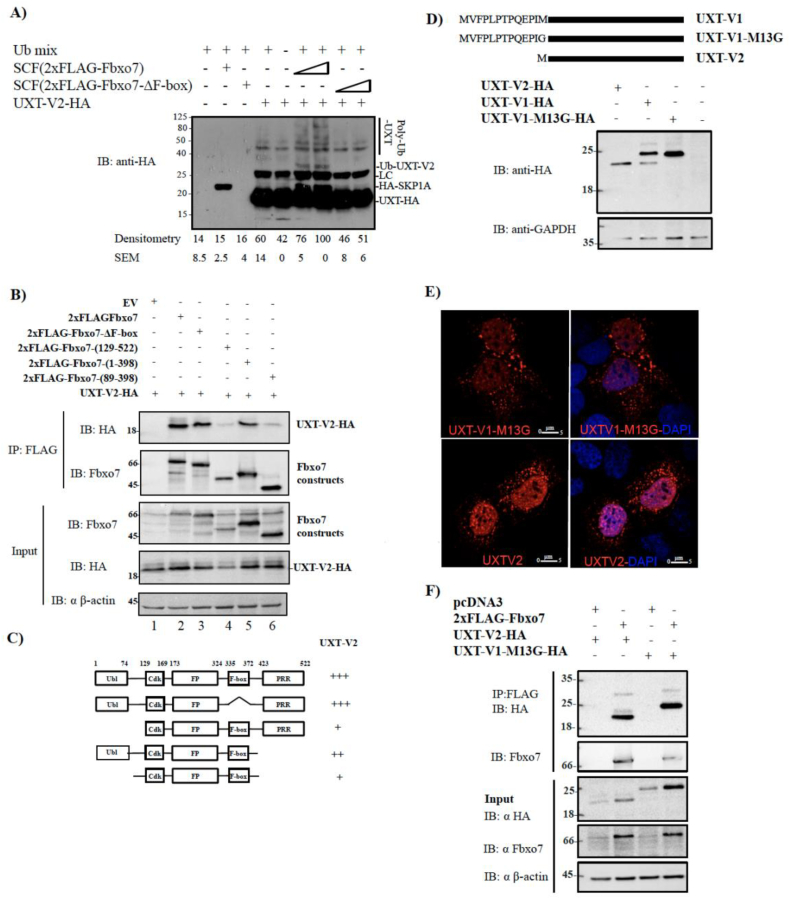
Fbxo7 ubiquitinates UXT-V2 *in vitro* and interacts with UXT-V2 and UXT-V1 *in vivo*. A) *In vitro* ubiquitination assays using purified SCF(2xFLAG-Fbxo7) or 2xFLAG-Fbxo7 lacking the F-box domain in two different concentrations (25 and 50 nM) and purified UXT-V2 from HEK293T cells as a substrate. The samples were used for western blotting, and an anti-HA antibody was used to visualize polyubiquitinated UXT—V2. The smear of each band was quantified by densitometry using Image J and the +/− SEM of the triplicates was calculated by GraphPad Prism; LC (light chain). B) Extracts of HEK293T cells transfected with the indicated 2xFLAG-Fbxo7 plasmids and UXT-V2-HA were immunoprecipitated with agarose anti-FLAG. Input and eluted proteins were subjected to immunoblotting with the indicated antibodies. C) Summary of the interaction mapping between UXT-V2 and Fbxo7 proteins. D) Representation of UXT—V1, UXT-V1-M13G and UXT-V2 isoforms and their expression in HEK293T cells transfected with each plasmid. E) Multiphoton microscopy of U2OS transfected with UXT-V1-M13G-HA or UXT-V2-HA. The slides were incubated with anti-HA and nuclei were probed by DAPI. F) HEK293T cells were transfected with FLAG-Fbxo7 and UXT-V1-M13G or UXT—V2. The cellular extracts were immunoprecipitated, resolved by SDS-PAGE and probed with the indicated antibodies. All the inputs represent 3% of the total protein used in coimmunoprecipitation assays. All cell lysates were obtained by NP-40 lysis buffer.

To validate the identification of UXT-V2 as a substrate of SCF(Fbxo7) from the protein microarray study described in [26], we performed an *in vitro* ubiquitination assay with purified SCF(Fbxo7) or mutant Fbxo7-ΔF-box E3 ligases at two different concentrations, using purified UXT-V2-HA from HEK293T cells as a substrate. The Fbxo7-ΔF-box mutant is unable to form an E3 ligase because it lacks the F-box domain that interacts with SKP1, and thus serves as a negative control. The reactions were performed in the presence of a ubiquitin mix composed of E1, E2, ubiquitin, ubiquitin buffer and ATP. A concentration dependent smear of polyubiquitinated UXT-V2 was observed only in the presence of SCF(Fbxo7), showing the specificity of the *in vitro* ubiquitination assays (Fig. 1A).

To map the interaction site on Fbxo7 for UXT—V2, HEK293T cells were co-transfected with plasmids encoding C-terminal HA-tagged UXT-V2 and N-terminal 2xFLAG-Fbxo7 or various mutants deleting specific domains, either singly or in combination. These included the Ubl domain (1–88), a linker region (89–128), the proline-rich region (PRR) (399–522) and the F-box domain (335–372). The F-box domain deletion did not affect the interaction of Fbxo7 with UXT-V2 (lane 3, Fig. 1B), indicating that this mutant maintain its substrate binding domain, as also observed previously [26]. However, deletions at the N-terminus of Fbxo7 removing the Ubl and linker region substantially reduced its interaction with UXT-V2 (lanes 4 and 6, Fig. 1B). These results, summarized in Fig. 1C, suggest that Ubl domain contributes to the interaction of Fbxo7 with UXT—V2.

In addition to isoform 2, another splice variant, UXT—V1, which is 12 amino acid residues longer than UXT-V2 at the N-terminus, has been described [27,36] (Fig. 1D). We observed that UXT-V1 plasmid also produced UXT-V2 isoform through translation at a second methionine at codon 13, ATG2. To bypass this issue, we obtained an UXT-V1 version with glycine substitution at ATG2 to produce UXT-V1-M13G, which prevented expression of UXT-V2 (Fig. 1D). To analyze the cellular distribution of both UXT isoforms, we carried out Multiphoton microscopy in U2OS cell lines transfected with UXT-V2-HA or UXT-V1-M13G-HA. We observed that both isoforms are present in the cytosol and cell nuclei, with UXT-V1-M13G being predominantly cytoplasmic and UXT-V2 within nuclei (Fig. 1E). We notice a punctate pattern in the cytoplasm upon transient expression of UXT-V1-M13G-HA and UXT-V2-HA. Punctate expression has been previously noted upon overexpression of UXT-V2 [37,38].

These isoforms have different functions and cellular localization, so we evaluated the *in vivo* interaction of both with Fbxo7, using co-immunoprecipitation assays. We observed that, similar to UXT—V2, UXT-V1-M13G also interacted with Fbxo7 (Fig. 1F), suggesting that both isoforms are potential substrates of SCF(Fbxo7).

### 3.2 Fbxo7 mediates proteasomal degradation of UXT-V2 but not UXT-V1-M13G

**Fig. 2:**
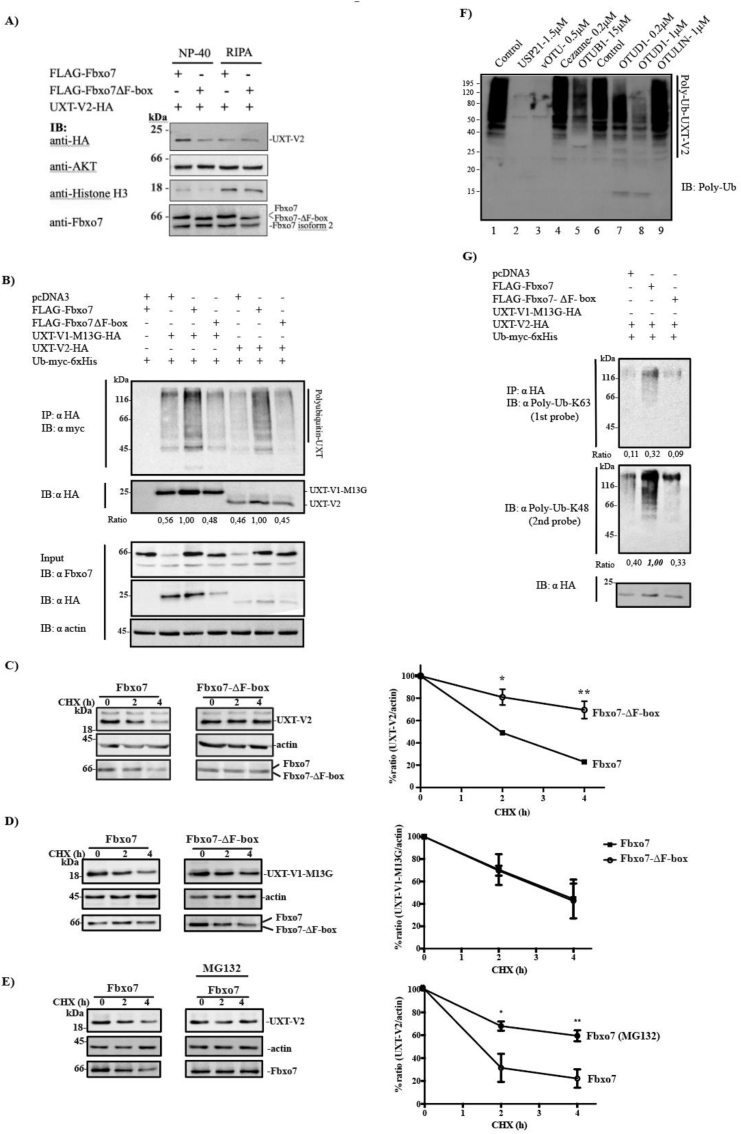
SCF(Fbxo7) ubiquitinates *in vivo* both isoforms of UXT and promotes proteasomal degradation of UXT—V2. A) UXT-V2 levels in cellular extracts obtained by cell lysis with NP-40 or RIPA buffer. HEK293T cells were transfected with indicated plasmids and the pellets were lysed with NP-40 or RIPA buffer. Total protein lysates (40 μg) were loaded in gel and the levels of UXT-V2 co-expressed with Fbxo7 or Fbxo7-ΔF-box were analyzed. This blot is representative of a triplicate experiment. B) HEK293T cells were transfected with the indicated plasmids, and the total cell lysates were immunoprecipitated with anti-HA. The HA peptide-eluted fractions were resolved by SDS-PAGE and used for western blot analyses with the indicated antibodies. The ratio between densitometry of the each smear and the corresponding anti-HA band is indicated. C) U2OS cells were transfected with Fbxo7 or Fbxo7-ΔF-box in combination with UXT-V2 or UXT-V1- M13G (D) and treated (2 or 4 h) with cycloheximide (CHX) for indicated times. Protein extracts were separated by SDS-PAGE, and the immunoblots were probed with the indicated antibodies. The quantitative analysis of the bands was performed by Image J and is shown in the graph. E) Similarly, CHX chase assay was performed in the presence or absence of the proteasome inhibitor MG132. The graph below shows the densitometric analysis by ImageJ of each protein band revealed by the indicated antibodies. GraphPad Prism was used to statistical analysis 2way ANOVA (Bonferroni posttest) Assays were performed in biological triplicate. *: *p* ≤ 0.05 and ** *p* ≤ 0.01. F) Purified DUBs were used to map polyubiquitinated UXT-V2 purified from HEK293T cells. The nonspecific DUBs (USP21 and vOTU) cleaved all ubiquitin chains; OTUB1 (K48-specific) partially removed ubiquitin chains and OTUD1 (preference for K63 linkages) showed concentration-dependent activity against the substrate. G) A fraction of the eluted proteins from A was subjected to western blot analysis with anti-K48 or anti-K63 specific antibodies. The ratios indicate the densitometry of each smear relative to the anti-HA band for a single experiment.

To investigate the functional consequences of polyubiquitination of UXT-V2 and UXT-V1-M13G by Fbxo7, we carried out a cycloheximide (CHX) chase analysis to evaluate the substrate stabilities *in vivo*. Different from previous cell lysis performed in Figs. 1B, F and 2A where we used NP-40 lysis buffer because of co-immunoprecipitation experiments, in CHX assays we lysed cells with RIPA buffer. The NP-40 lysis buffer did not lyse the cell nuclei completely compared to RIPA buffer, as can be seen in the immunoblots for the nuclear marker histone H3, which is clearly higher when RIPA was used (Fig. 2A). Similar to Fig. 2C, it is also evident that using NP-40 buffer results in higher UXT-V2 levels when co-expressed with Fbxo7 compared to Fbxo7-ΔF-box. However, the opposite result was obtained when RIPA was utilized, *i.e.* UXT-V2 expression is higher in the presence of Fbxo7-ΔF-box (Fig. 2A).

Since both UXT-V1-M13G and UXT-V2 interact with Fbxo7 and UXT-V2 were confirmed as substrate through *in vitro* ubiquitination assay, we performed *in vivo* ubiquitination assays in HEK293T cells to evaluate whether they are both substrates of SCF(Fbxo7). Cells were transfected with plasmids encoding C-terminal HA-tagged UXT-V1-M13G or UXT-V2 in combination with ubiquitin-myc and Fbxo7 or the negative controls peGFP-N1 and the ligase dead mutant, Fbxo7-ΔF-box. Therefore, although the Fbxo7-ΔF-box mutant interacts with UXT—V2, it is unable to ubiquitinate Fbxo7 substrates. The proteasome inhibitor MG132 was used to allow accumulation of poly-ubiquitinated proteins in the cells, and immunoprecipitation with agarose anti-HA was carried out. A strong smear signal of poly-ubiquitinated protein was seen when Fbxo7 was co-expressed with UXT-V2 and UXT-V1-M13G confirming that both isoforms of UXT are SCF(Fbxo7) substrates (Fig. 2B).

U2OS cells were transfected with plasmids encoding UXT-V2-HA or UXT-V1-M13G-HA and 2xFLAG-Fbxo7 or 2xFLAG-Fbxo7-ΔF-box. The cells were either left untreated or incubated for 2 or 4 h with CHX, and the levels of UXT-V2-HA in the lysates were evaluated. UXT-V2 levels were reduced to 50% of their initial amount after 2 h of treatment when co-expressed with Fbxo7, and only 20% of the protein remained after 4 h (Fig. 2C). By contrast, even after 4 h of treatment, 80% of UXT-V2 was present in cell lysates when Fbxo7-ΔF-box was co-expressed (Fig. 2C). These results indicate that Fbxo7 decreases the stability of UXT-V2 in an F-box domain-dependent manner. On the other hand, the reduction of UXT-V1-M13G levels after 2 and 4 h of treatment was comparable between WT and mutant Fbxo7, indicating that its degradation was not mediated by Fbxo7 (Fig. 2D). To prove that UXT-V2 polyubiquitination directs its degradation by the proteasome we performed a CHX assay on Fbxo7-transfected cells in the presence of the proteasome inhibitor MG132. Strikingly, MG132 treatment inhibited UXT-V2 degradation by the proteasome when co-expressed with Fbxo7 WT (Fig. 2E), confirming that this ubiquitin-ligase promotes the proteasomal degradation of UXT-V2 in cells.

The linkage type of the polyubiquitin chains added to substrates by E3 ubiquitin ligases determines their fate in the cell [39]. K48 poly-ubiquitin chains often regulate protein stability by directing the substrate to the proteasome, while the K63 chain is a nondegradative modification related to protein regulation [7]. To determine the type of ubiquitin chains added by SCF(Fbxo7) to UXT—V2, we applied ubiquitin chain restriction analysis using deubiquitinating enzymes (DUBs) called UbiCRest [34]. We used UXT-V2 obtained by immunoprecipitating UXT-V2-HA from cells transfected with Fbxo7, as shown in Fig. 2B (lane 6). Polyubiquitinated UXT-V2 was treated with a panel of specific DUBs, including two positive controls, USP21 and vOTU, which are nonspecific DUBs that are capable of cleaving all types of ubiquitin chains containing isopeptide bonds (lanes 2 and 3, Fig. 2F). Treatment with the K48-specific DUB OTUB1 reduced the high molecular weight smear intensity compared to the negative control (lanes 5 and 1, Fig. 2F). Additionally, a diminished intensity of the poly-ubiquitinated UXT-V2 smear was observed when increasing concentrations of the K63- selective OTUD1 were applied (lanes 7 and 8, Fig. 2F), while enzymes targeting K11 (Cezanne) and M1 (OTULIN) linkages had no effect. To confirm the polyubiquitin chain types present on UXT-V2-HA *in vivo*, we probed the *in vivo* ubiquitinated samples with anti-K48 or anti-K63 specific antibodies. Strikingly, we observed both polyubiquitin chains in UXT-V2-HA confirming the results of UbiCRest assay (Fig. 2G). Taken together, these data shows that Fbxo7 promotes proteasomal degradation of UXT-V2 through introduction of K48 polyubiquitin chains. It remains to be determined the nature of polyubiquitin chain introduced in UXT-V1-M13G-HA and the function of K63-linked ubiquitin chains in UXT—V2.

### 3.3 *FBXO7* knockdown and Fbxo7-ΔF-box expression promote UXT-V2 accumulation

**Fig. 3:**
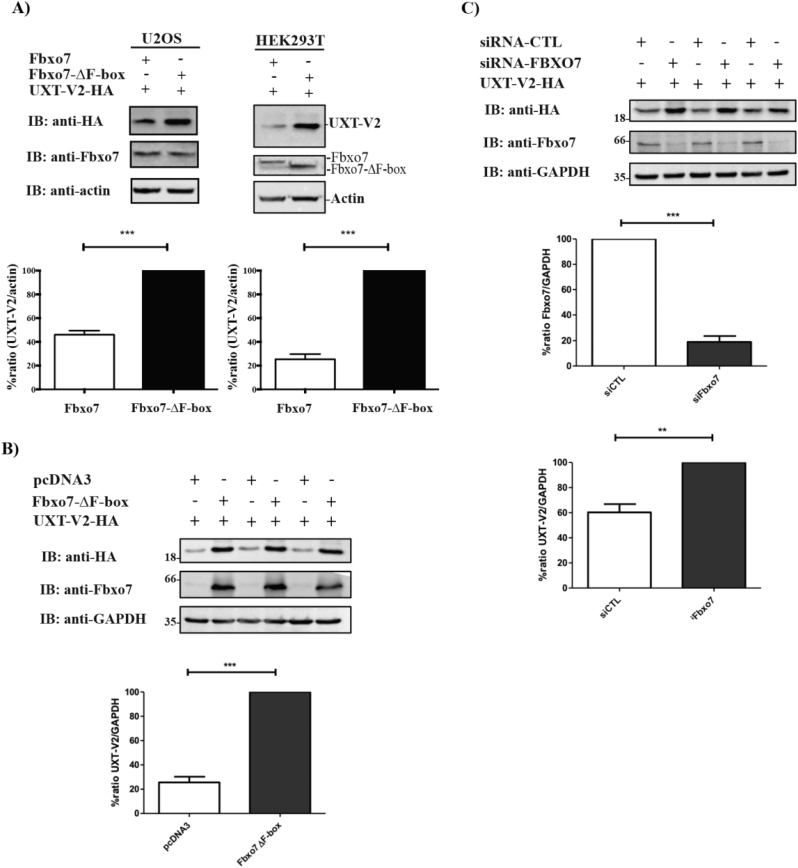
UXT-V2 is a substrate of Fbxo7. A) HEK293T or U2OS cell lines were transfected with indicated plasmids, and cell lysates were immunoblotted with indicated antibodies. The graphs show the densitometric analysis of the UXT/actin ratio. B) HEK293T cells were co-transfected with empty vector or Fbxo7-ΔF-box in the presence of UXT-V2-HA and total cell lysates were used to western blotting probed with indicated antibodies. C) U2OS cells were transfected with siRNA control or siRNA to *FBXO7*. After 48 h, the cell lysates were resolved in SDS-PAGE and immunoblotted with the indicated antibodies. The densitometry was carried out by ImageJ and GraphPad Prism was used to plot the graphs and statistical analysis (Student's *t*-test). All the assays were performed in triplicate and cells were lysed with RIPA buffer. *: *p* ≤ 0.05 and **: *p* ≤ 0.01.

To explore the effect of Fbxo7 expression on UXT-V2-HA protein levels in different cell lines, HEK293T or U2OS were co-transfected with 2xFLAG-Fbxo7 or 2xFLAG-Fbxo7-ΔF-box and UXT-V2-HA, and the total cell lysates were analyzed by western blot. When UXT-V2 was co-expressed with Fbxo7-ΔF-box, we observed an accumulation of this substrate when compared to WT Fbxo7 in both cell lines (Fig. 3A). These results suggest that Fbxo7-ΔF-box interact with UXT-V2-HA and prevented its ubiquitin-mediated degradation by Fbxo7. To show the dominant negative effect of Fbxo7-ΔF-box in UXT-V2-HA, we co-transfect cells with empty vector or Fbxo7-ΔF-box in the presence of UXT-V2-HA. Strikingly, Fbxo7-ΔF-box promoted an accumulation of UXT-V2-HA compared to empty vector (Fig. 3B). To confirm that UXT-V2-HA levels were specifically controlled by Fbxo7, we knocked down *FBXO7* and evaluate UXT-V2-HA levels in total cell lysates. We observed an accumulation of UXT-V2-HA compared to the siRNA control (Fig. 3C), suggesting that Fbxo7 regulates UXT-V2-HA protein levels by promoting its degradation.

### 3.4 UXT-V2 colocalizes with Fbxo7 into the cell nucleus

**Fig. 4:**
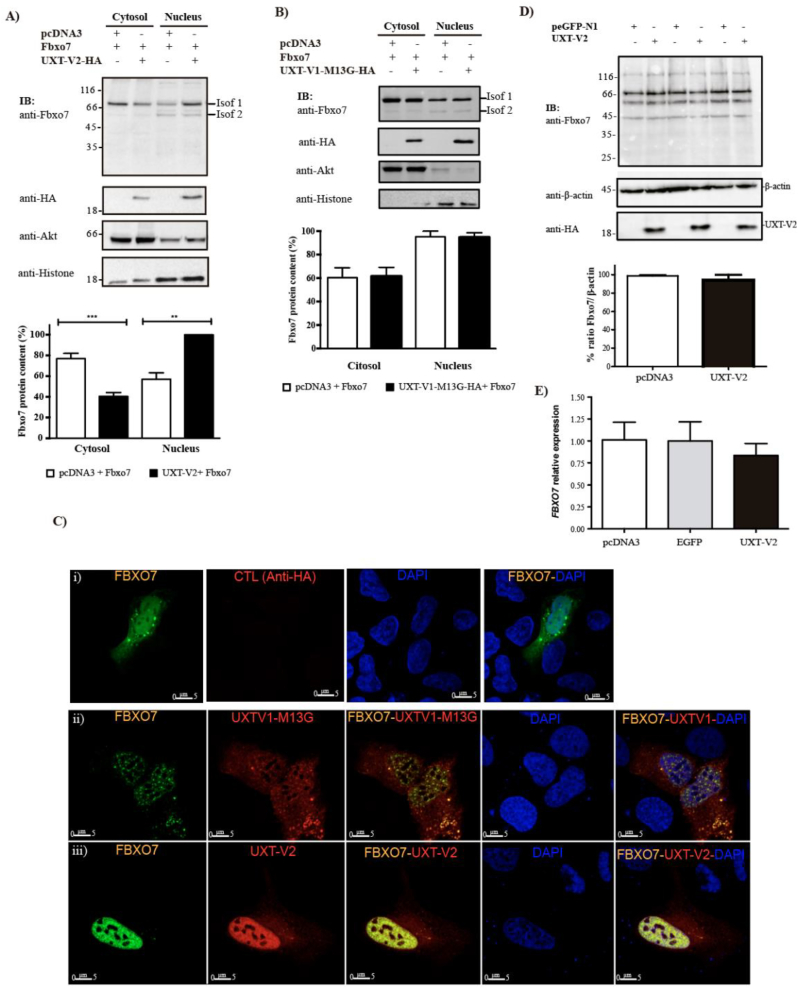
UXT-V2 overexpression increases increases Fbxo7 levels levels into the cell nucleus. A and B) U2OS cells were transfected with the indicated plasmids, and the cytosolic- and nuclear-enriched fractions were resolved by SDS-PAGE and analyzed by western blotting with the indicated antibodies. Histone and Akt were probed as nuclear and cytoplasmic markers, respectively. GraphPad Prism was used to statistical test (Student's *t*-test), and all assays were performed in triplicate. **: *p* ≤ 0.05 and ***: *p* ≤ 0.01. C) U2OS cells were transfected with (i) Fbxo7, (ii) Fbxo7 and UXT-V1-M13G or (iii) Fbxo7 and UXT—V2. The slides were incubated with anti-HA or anti-Fbxo7 and visualized by goat anti-rabbit-Alexa 488 or donkey anti-mouse-Alexa 594. Nuclei were stained with DAPI and an objective of 63× was used. D) HEK293T cells were transfected with pEGFP-N1 or UXT-V2-HA, and total cell lysates were resolved by SDS-PAGE and immunoblotted with the indicated antibodies. The Fbxo7 protein levels are presented in the graph with densitometry values obtained by ImageJ. E) mRNA was purified from HEK293T cells transfected with pcDNA3, UXT-V2-HA or pEEGFP-N1. RT-qPCR analysis was carried out for *FBXO7* expression and evaluated in comparison to the *GAPDH*. No significant differences were found among the groups (*n* = 3) by using one-way ANOVA (*p value* = 0.4807).

Fbxo7 has been reported to be localized in the nucleus when fused with dsRED at its C-terminus [14] or to both the nucleus and cytoplasm when N-terminally tagged with a FLAG-epitope [40]. The localization of FLAG-Fbxo7 depends on the cell cycle phase, being cytoplasmic during G0/G1 and accumulating in the nucleus as cells transition into S/G2 phase [40]. In human dopaminergic SK-N-SH cells, overexpressed Fbxo7 forms protein aggregates in the cytoplasm, and this effect is time- and stress-dependent [22]. To investigate the interaction between Fbxo7 and UXT-V2 at a subcellular level, we evaluated the effect of UXT-V2 expression on the localization of Fbxo7. We performed a cellular fractionation with cells overexpressing UXT-V2 or a control plasmid and assayed Fbxo7 levels in each cell compartment by immunoblotting. We found UXT-V2 expression increased the levels of Fbxo7 in the nuclear fraction (Fig. 4A). To confirm that this effect is specific for UXT—V2, we evaluated the localization of Fbxo7 upon overexpression of UXT-V1-M13G. Notably, the expression of this longer UXT isoform did not alter the subcellular distribution of Fbxo7 (Fig. 4B). To further test the opposing effects of both UXT isoforms on Fbxo7 localization, we performed a Multiphoton microscopy of U2OS cells co-transfected with Fbxo7 and either UXT-V1-M13G or UXT—V2. Consistent with previous studies [38], Fbxo7 is distributed in the cytosol and nucleus (Fig. 4 Ci). When Fbxo7 was co-expressed with UXT-V1-M13G, we observed that Fbxo7 adopted a dotted distribution in cell nuclei (Fig. 4 Cii). On the other hands, Fbxo7 colocalizes with co-expressed UXT-V2 into the cell nucleus, exhibiting a predominantly nuclear localization (Fig. 4 Ciii), compared to Fig. 4 Ci. These results suggest two alternative hypotheses: UXT-V2 increases the total levels of Fbxo7 or promotes its nuclear accumulation. However, overexpressing UXT-V2 did not affect Fbxo7 protein or mRNA levels in HEK293T cells (Figs. 4D and E), indicating that UXT-V2 promotes the nuclear localization of Fbxo7.

### 3.5 Fbxo7 inhibits the NF-κB signaling pathway

**Fig. 5:**
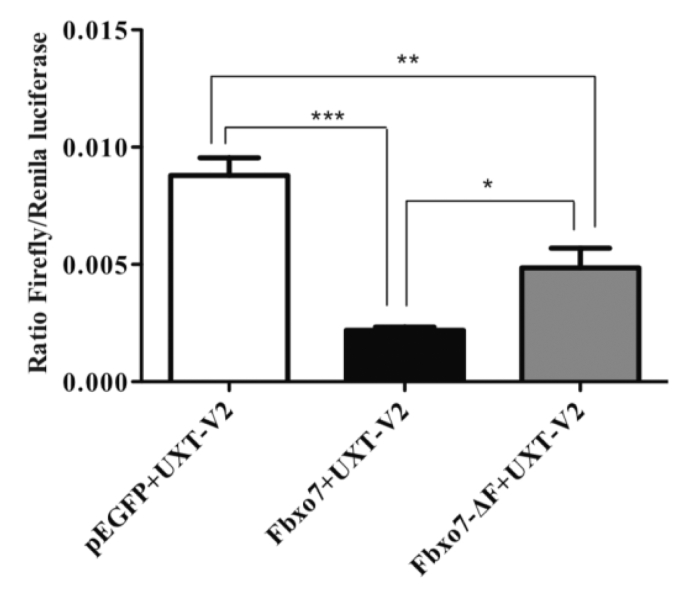
Fbxo7 impairs the NF-B signaling pathway. HEK293 cells stably expressing the NF-κB-luciferase reporter gene were transfected with the indicated plasmids along with a Renilla luciferase reporter as a transfection control. After 24 h of transfection, the cells were stimulated with TNF-α during serum starvation and harvested. After 48 h, cells were harvested and luciferase expression was assayed in quintuplicate from cell lysates and luciferase levels were normalized to Renilla levels. *: *p* < 0.05, **: *p* *=* 0.01, ***; *p* < 0.001. One-way ANOVA, posttest Newman-Keuls. *n* = 5 (GraphPad Prism).

UXT-V2 is an essential component of the NF-κB enhanceosome in the nucleus, interacting with p65 and modulating the TNF-α response [28]. UXT-V2 also interacts with EZH1 and SUZ12 in the nucleus to synergistically regulate this pathway [41]. *UXT-V2* knockdown, but not its overexpression, impairs the transcriptional activation of NF-κB target genes induced by TNF-α [28,41]. To evaluate the consequences of UXT-V2 degradation by Fbxo7 on the NF-κB pathway, we transfected HEK293 cells stably expressing a reporter gene for the NF-κB pathway fused to firefly luciferase. UXT-V2 was transfected in combination with pEGFP-N1, Fbxo7 or Fbxo7-ΔF-box mutant, and the cells were stimulated by TNF-α. Strikingly, Fbxo7 decreased NF-κB pBIIx reporter gene levels compared to UXT-V2 with pEGFP or UXT-V2 in combination with Fbxo7-ΔF-box (Fig. 5). These results are consistent with the proteasomal degradation of UXT-V2 mediated by Fbxo7 and consequently inhibition of NF-κB.

## 4 Discussion

Different approaches for E3 ubiquitin ligase substrate identification have been developed, including *in vivo* protein partner searching, such as by co-immunoprecipitation and mass spectrometry, two-hybrid screening and protein microarrays [42]. Previously, we reported the identification of 338 new Fbxo7 substrates by using *in vitro* ubiquitination of protein microarrays, and one of these targets was the UXT-V2 protein. Since Fbxo7 and UXT-V2 are involved in similar processes, such as cellular transformation and NF-κB signaling regulation [14,23,28,29], we investigated the relationship between these proteins.

The Protoarrays® are composed of proteins immobilized *via* the N- or C-terminus to a nitrocellulose-activated surface, which can alter the UXT-V2 structure, forcing the interaction with Fbxo7. To bypass this constraint, we mapped the *in vivo* interaction between these proteins through co-immunoprecipitation assays. In addition to the F-box domain (residues 335–372), Fbxo7 is composed of four other characterized domains: Ubl (ubiquitin-like; 1–74), Cdk-binding (129–169), FP (Fbxo7/PI31; 173–224) and a PRR (proline-rich region; 423–522). The Ubl domain is responsible for the Fbxo7 interaction with TOMM20 and GSK3β [26], and we observed that the Ubl domain also contributes to Fbxo7 interaction with UXT—V2. To validate the direct ubiquitination of UXT-V2 by SCF(Fbxo7), we carried out an *in vitro* ubiquitination assay with UXT-V2 and SCF(Fbxo7) complexes purified from HEK293T cells. We observed that UXT-V2 was only poly-ubiquitinated by wild-type Fbxo7, confirming it as a *bona fide* substrate of this E3 ligase. It has been reported that Epstein-Barr virus (EBV) BGLF4 kinase phosphorylates UXT-V2 at Thr3, a modification which reduces its interaction with NF-κB transcription factors and decreases NF-κB enhanceosome activity [43]. It is possible that the phosphorylation of UXT-V2 may also promote Fbxo7-mediated ubiquitination and degradation, although we did not test for this. To date no mammalian kinases have been reported to phosphorylate UXT—V2, but we cannot exclude that post-translation modification of UXT-V2 mediates its interaction with and ubiquitination by SCF(Fbxo7).

Another splice variant of UXT called UXT-V1 that is 12 amino acids longer than UXT-V2 at the N-terminus, where it binds to TRAF2 has been studied. It isis a short-lived cytoplasmic protein that protects cells against TNF-induced apoptosis through its interaction with TRAF2, which prevents the TRAF2-RIP-TRADD complex from recruiting FADD and caspase 8 [36]. Because of the high structural identity with UXT—V2, we evaluated the interaction of UXT-V1 with Fbxo7. The plasmid encoding UXT-V1 also expressed UXT-V2 due to an alternative internal ATG codon at position 37. Thus, we obtained an UXT-V1-M13G with a mutation at the second ATG, which only produce the isoform 1. This clone obviates UXT-V2 expression from the plasmid. We evaluated the localization of UXT-V2-HA and UXT-V1-M13G-HA in U2OS cells transfected with these plasmids using Multiphoton microscopy. Our results differ from the described by Huang et al. [29]; however some differences in the choice and placement of fusions to enable detection between our studies could account for this, including our study of the only UXT-V1-M13G variant; we used an HA tag at the C-terminus and the Huang, et al. used an N-terminal GFP fusion. This results in proteins with different molecular weights, with UXT-V1-M13G-HA (~20 kDa) and GFP-UXT-V1 (~45 kDa). We also confirmed that isoform 1 interacts with Fbxo7. In summary, our interaction assays indicate that both UXT-V1 and UXT-V2 interact with Fbxo7, indicating that the TRAF2 binding site of UXT-V1 [36] is not essential for this interaction.

To verify that the ubiquitination of UXT-V2 by SCF(Fbxo7) reported in [26] also occurs in the cells, we performed an *in vivo* ubiquitination assay. Moreover, we compared the ubiquitination of UXT-V1-M13G and UXT—V2, to independently assess the ability of each isoform to be ubiquitinated, since there are no lysine residues inside the additional 12 amino acid sequence of UXT—V1. Importantly, both isoforms of UXT were ubiquitinated by SCF(Fbxo7), indicating that the TRAF2 binding domain of UXT-V1 is not required for ubiquitination of UXT-V1 by Fbxo7.

Polyubiquitination is a reversible post-translational modification that regulates many biological processes such as cell-cycle progression, DNA repair, apoptosis, receptor endocytosis [39]. Ubiquitin can be attached to a substrate either as a monomer or as a polyubiquitin chain with different lengths and linkage types. The distinct conformational structure that ubiquitin chains adopt determines their different functions [7]. To evaluate the functional relevance of UXT-V1 and UXT-V2 ubiquitination by Fbxo7, we evaluated the stability of these proteins in the presence of Fbxo7 or Fbxo7-ΔF-box through CHX assays. We showed that Fbxo7 modulates the abundance of UXT-V2 in cells, decreasing its half-life when Fbxo7 wild-type was co-expressed. In contrast, we observed that degradation of UXT-V1-M13G was independent of E3 ligase function of Fbxo7, once no differences were evident when Fbxo7-ΔF-box was used. It worth to mention that RIPA buffer was utilized in these assays once we observed that NP-40 buffer used in co-IP assays did not disrupt cell nuclei. Interestingly, the levels of UXT-V2 decreased when co-expressed with Fbxo7 compared to Fbxo7-ΔF-box in cell lysates with RIPA buffer, and the contrary was observed when NP-40 buffer was used. Thus, as the translation occurs in the cytoplasm and UXT-V2 is being degraded by Fbxo7 WT, the mechanism to replace the UXT-V2 is increasing its levels, which is observed predominantly in the cytosol. However, when RIPA buffer is used to efficiently lyse nuclei, we observe lower levels of UXT-V2 in the presence of Fbxo7.

To characterize the ubiquitin chain added to this substrate we carried out UbiCRest assay and used specific antibodies against K48 and K63 linkages. The UbiCRest assay showed that UXT-V2 was modified by both K48 and K63 polyubiquitin chains but did not exclude the possibility for other linkage types. By using chain-specific antibodies, we confirmed both Ub-chain types on UXT—V2. The K48 modification is associated with the proteasomal degradation of substrates, and we confirmed that Fbxo7 modulates the abundance of UXT-V2 in cells, decreasing its half-life when Fbxo7 wild-type was co-expressed by CHX assays. The knockdown of *FBXO7* increased UXT-V2 levels in the cells, and the co-expression of Fbxo7-ΔF-box with UXT-V2 in two different cell lines, promoted its accumulation as compared to its co-expression with wild-type Fbxo7. This suggests that mutant Fbxo7 protects UXT-V2 from degradation and we confirmed its dominant negative effect when co-expressed with UXT-V2-HA compared to empty vector. Overall, these results show UXT-V2 is a canonical substrate of Fbxo7.

It was reported that a tumor suppressor protein lysyl oxidase proenzyme propeptide region (LOX-PP) interacts with UXT-V2 and relocalizes this substrate to the cytoplasm, decreasing its stability [44]. Since most of the catalytically active proteasomes are localized in the cytoplasm [45], we speculate that polyubiquitinated UXT-V2 is translocated to the cytosol for degradation by the proteasome. Whether LOX-PP is the protein responsible for mediating this process remains to be evaluated in future studies.

We also observed the some K63 ubiquitin chains on UXT—V2, which is a proteasome-independent modification related to signal transduction regulation. Recently, it was described that K63 chains are seeds for K48/K63 branched ubiquitin chains that mediate substrate degradation. The K63 chain in the proapoptotic regulator TXNIP introduced by the E3 ligase ITCH triggers the assembly of K48/K63 branched chains by recruiting the ubiquitin-interacting ligase UBR5, leading to proteasomal degradation of TXNIP [46]. Fbxo7 has a ubiquitin-like domain at the N-terminus, which is important for its interaction with UXT-V2 and other described substrates (TOMM20 and GSK3β), but it remains unknown whether Fbxo7 mediates K63/K48 branched polyubiquitin or whether it functions as a K63-interacting E3 ligase to promote the addition of the K48 ubiquitin chains to this substrate.

Fbxo7 has a functional leucine-rich nuclear export sequence (NES) embedded within the F-box domain that binds to exportin 1 (CRM1), allowing Fbxo7 to migrate to the nucleus. The interaction with CRM1 competes with SKP1 inhibiting SCF(Fbxo7) formation, and this competitive mechanism allows Fbxo7 to be present in different cellular compartments [40]. Since UXT-V2 is a predominantly nuclear protein, we evaluated whether it mediates Fbxo7 accumulation to the nucleus. Our fractionation data demonstrated that UXT-V2 overexpression increased the Fbxo7 content in the nucleus, suggesting that the functional relevance of the interaction between these proteins is associated with a nuclear process. In fact, the Multiphoton microscopy showed that the subcellular distribution of Fbxo7 changed when co-expressed with UXT—V2, colocalizing with its substrate in the nucleus. The specificity of this effect was demonstrated by UXT—V1, where no changes in Fbxo7 distribution were observed when these proteins were co-expressed. We cannot exclude the possibility that UXT-V2 overexpression stabilizes Fbxo7 in the cell nucleus either by preventing Fbxo7 association with the nuclear export machinery or by preventing its autoubiquitination.

Fbxo7 has opposing functions in the NF-κB signaling pathway depending on the receptor involved. While Fbxo7 positively regulates formation of the BMPR–NRAGE–TAK1–TAB1 complex up-regulating NF-κB activity [25], it also negatively regulates this pathway by ubiquitinating TRAF2 and cIAP-1, which decreases RIP1 ubiquitination and diminishes NF-κB signaling [23]. Since UXT-V2 is a component of the NF-κB enhanceosome in cell nuclei, and its knockdown decreases TNFR activation of this pathway [28], we explored the role of Fbxo7 through this receptor. We showed that Fbxo7 decreased the activation of NF-κB signaling in the presence of UXT—V2, whereas overexpression of this substrate with pEGFP restored the activation of this pathway. When compared the co-transfection of UXT-V2 with WT Fbxo7 or with Fbxo7-ΔF-box, we observed an increase in NF-κB signaling by the Fbxo7-ΔF-box mutant, suggesting that it interacts with and protects UXT-V2 from degradation caused by Fbxo7, promoting the activation of the NF-κB signaling pathway. However, Fbxo7-ΔF-box also inhibits the activation of this signaling compared to the control. It has been demonstrated that Fbxo7-ΔF-box predominantly localizes in the nucleus, and we observed a stable interaction between Fbxo7-ΔF-box and UXT-V2 in co-IP assays. We hypothesize that the interaction between these proteins could sequester UXT-V2 impairing its capacity to assemble the enhanceosome and consequently reducing the activation of NF-κB genes. It has been shown that polyubiquitin chains branched at K48 and K63 regulate NF-κB signaling. The E3 ligase HUWE1 generates K48 branches on K63 chains formed by TRAF6, and these K48-K63 branched chains recruit TAB2, which protects the K63 linkages from CYLD-mediated deubiquitination, thereby amplifying NF-κB signals [46]. It remains to be investigated whether K63 and K48 polyubiquitination of UXT-V2 by Fbxo7 forms a branched ubiquitin chain involved in NF-κB signaling regulation. In summary, we have described a new mechanism of NF-κB signaling pathway inhibition through the ubiquitination and degradation of UXT-V2 by the E3 ubiquitin ligase SCF(Fbxo7).

## Data availability

All data generated or analyzed during this study are included in this published article.

## Author contribution

Investigation, methodology, formal analysis, validation: V.S., C.A.B.O., S.J.R., T.E.T.M. and F.R.T.. Methodology: P.M.S.P., C.R.S.T.B·C., A.C.M. and J.S·O.. Supervision: F.R.T., H.L, M.D.G., D.K.. Fundind acquisition: F.R.T., M.D.G., H.L. Project administration: F.R.T.. Writing-original draft: V.S., C.A.B.O., F.R.T. Writing-review editing: F.R.T., V.S., H.L., M.D.G., T.E.T.M.

## Funding

V.S. was funded by a FAPESP 2017/07879-9; C.A.B.O. was funded by FAPESP 2016/21310-6; N.B·S is funded by FAPESP 2017/22153-4; J.S·O is funded by FAPESP 2018/09204-1, C.R.S.T.B.C was funded by a PIBIC/CNPq UFSCAr scholarship and FAPESP 2019/03943-0; P.M.S.P. is funded by CAPES scholarship PPGGEv/UFSCar; T.E.T.M. was funded by the Marie Curie ITN ‘UPStream’; A.C.M. is funded by CAPES scholarship. M.D.G was funded by FAPESP 2018/01308-2 and FAEPA/FMRP-USP; D.K. was supported by the 10.13039/501100000265Medical Research Council [U105192732], the 10.13039/501100000781European Research Council [724804], and the Lister Institute for Preventive Medicine; S.J.R. and H.L. were funded by the Biotechnology and Biological Science Research Council [BB/J007846/1]; F.R.T. was funded by FAPESP 2016/00792-2, FAPESP 2016/25798-3 and CAPES.

## Declaration of Competing Interest

The authors declare no competing interests associated with this manuscript.
